# Ultrasound Assessment of Hepatic Steatosis by Using the Double Nakagami Distribution: A Feasibility Study

**DOI:** 10.3390/diagnostics10080557

**Published:** 2020-08-04

**Authors:** Feng Fang, Jui Fang, Qiang Li, Dar-In Tai, Yung-Liang Wan, Kazuki Tamura, Tadashi Yamaguchi, Po-Hsiang Tsui

**Affiliations:** 1 School of Microelectronics, Tianjin University, Tianjin 300072, China; fangfeng2018@tju.edu.cn (F.F.); liqiang@tju.edu.cn (Q.L.); 2 x-Dimension Center for Medical Research and Translation, China Medical University Hospital, Taichung 404332, Taiwan; juifang2014@gmail.com; 3 Department of Gastroenterology and Hepatology, Chang Gung Memorial Hospital at Linkou, Chang Gung University, Taoyuan 33305, Taiwan; tai48978@cgmh.org.tw; 4 Department of Medical Imaging and Radiological Sciences, College of Medicine, Chang Gung University, Taoyuan 33302, Taiwan; ylw0518@gmail.com; 5 Medical Imaging Research Center, Institute for Radiological Research, Chang Gung University and Chang Gung Memorial Hospital at Linkou, Taoyuan 33302, Taiwan; 6 Department of Medical Imaging and Intervention, Chang Gung Memorial Hospital at Linkou, Taoyuan 33305, Taiwan; 7 Preeminent Medical Photonics Education & Research Center, Hamamatsu University School of Medicine, Shizuoka 431-3192, Japan; k.tamura@hama-med.ac.jp; 8 Center for Frontier Medical Engineering, Chiba University, Chiba 263-8522, Japan

**Keywords:** fatty liver, hepatic steatosis, ultrasound, Nakagami distribution

## Abstract

Ultrasound imaging is a first-line assessment tool for hepatic steatosis. Properties of tissue microstructures correlate with the statistical distribution of ultrasound backscattered signals, which can be described by the Nakagami distribution (a widely adopted approximation of backscattered statistics). The double Nakagami distribution (DND) model, which combines two Nakagami distributions, was recently proposed for using high-frequency ultrasound to analyze backscattered statistics corresponding to lipid droplets in the fat-infiltrated liver. This study evaluated the clinical feasibility of the DND model in ultrasound parametric imaging of hepatic steatosis by conducting clinical experiments using low-frequency ultrasound dedicated to general abdominal examinations. A total of 204 patients were recruited, and ultrasound image raw data were acquired using a 3.5 MHz array transducer for DND parametric imaging using the sliding window technique. The DND parameters were compared with hepatic steatosis grades identified histologically. A receiver operating characteristic (ROC) curve analysis was used to evaluate the diagnostic performance. The results indicated that DND parametric imaging constructed using a sliding window with the side length of five times the pulse length of the transducer provided stable and reliable DND parameter estimations and visualized changes in the backscattered statistics caused by hepatic steatosis. The DND parameter increased with the hepatic steatosis grade. The areas under the ROC curve for identifying hepatic steatosis were 0.76 (≥mild), 0.81 (≥moderate), and 0.82 (≥severe). When using low-frequency ultrasound, DND imaging allows the clinical detection of hepatic steatosis and reflects information associated with lipid droplets in the fat-infiltrated liver.

## 1. Introduction

Hepatic steatosis, a condition in which excessive fat forms fatty vacuoles and accumulates in liver cells [1], may progress to nonalcoholic steatohepatitis, fibrosis, cirrhosis, and even hepatocellular carcinoma [2,3,4]. Nonalcoholic fatty liver disease (NAFLD) induced by hepatic steatosis has become the leading cause of chronic liver disease [5], and it is closely related to metabolic syndromes, including cardiovascular disease, obesity, diabetes, and dyslipidemia [3,6]. Therefore, the diagnosis and assessment of hepatic steatosis are essential to preventing the further deterioration of liver diseases.

Liver biopsy is the gold standard for diagnosing hepatic steatosis [7,8]; however, it is not suitable as a routine examination due to limitations, such as sampling error, invasiveness, and other complications [9]. Thus, noninvasive medical imaging has clinical significance for assisting in the quantitative diagnosis of hepatic steatosis. Computed tomography (CT) [10], magnetic resonance spectroscopy (MRS) [11], magnetic resonance imaging, and ultrasound are currently used for the imaging and analysis of hepatic steatosis [12,13]. Ultrasound B-mode imaging plays a comparatively first-line role in the clinical evaluation of hepatic steatosis due to its portability, cost-effectiveness, real-time capability, and nonionizing radiation [12,14,15]. However, ultrasound B-mode imaging based on the amplitude of the envelopes of beamformed radiofrequency (RF) signals is easily affected by system settings and user experience [16,17,18]. To achieve a relatively objective assessment of hepatic steatosis, the quantitative analysis of raw image data may provide valuable diagnostic clues.

Acoustically, the liver parenchyma may be modeled as microstructures consisting of considerable scatterers [19,20]. Acoustic scatterers interact with incident ultrasound to contribute ultrasound backscattered signals and form the corresponding speckle pattern in the ultrasound B-mode image; considering the randomness of ultrasound backscattering, the envelope statistics (i.e., the echo amplitude distribution), which depend on tissue microstructures, can be described by using the statistical distribution models for tissue characterization [21,22]. Homodyned-K distribution is the general model to encompass various backscattering conditions, including pre-Rayleigh, Rayleigh, and post-Rayleigh distributions [22]; its approximation is the Nakagami distribution, which has become the most frequently used model for tissue characterization because it provides a general description of the backscattered statistics with simplicity and low computational complexity [22]. The Nakagami parameter, which provides an estimation of the backscattered statistics, has been applied to ultrasound parametric imaging of hepatic steatosis, demonstrating that Nakagami imaging performs well in the assessment of the fatty liver [23,24,25].

Although the Nakagami parameter provides a quantitative description of practical changes in the echo amplitude distribution caused by hepatic steatosis, its physical meanings are ambiguous because of confounding information contributed by two scattering sources—normal and fat-infiltrated hepatocytes. To resolve this problem, the double Nakagami distribution (DND) has recently been proposed as a new model for describing the backscattered statistics of hepatic steatosis under high-frequency ultrasound excitation. The DND model combines two Nakagami distributions to allow the estimation of the Nakagami parameter corresponding to lipid droplets, and the feasibility of DND-based parametric imaging for detecting the fatty liver was explored through ex vivo animal experiments [26]. While proposing the DND model as a new approach to detect hepatic steatosis using high-frequency ultrasound, the diagnostic performance of DND imaging in clinical evaluations of the hepatic steatosis grade remains unknown.

In this study, we investigated the clinical feasibility of ultrasound DND imaging constructed using low-frequency ultrasound in the assessment of hepatic steatosis. Abdominal ultrasound examinations of patients were carried out for validation of the proposed method. The performance of ultrasound DND imaging in diagnosing hepatic steatosis was reported, and the clinical usefulness of DND imaging was discussed.

## 2. Materials and Methods

### 2.1. Double Nakagami Distribution

The probability density function (PDF) *f*(*x*) of the Nakagami statistical model for the ultrasonic backscattered envelope signal *x* is given by

(1) $$f\left(x\right)=\frac{2m^{m}x^{2m-1}}{\Gamma \left(m\right)\Omega^{m}}\exp(-\frac{m}{\Omega }x^{2})U\left(x\right)$$

where Γ(·) and U(·) are the gamma function and the unit step function, respectively. The scaling parameter Ω is related to the echo energy. The Nakagami parameter *m* is associated with the statistical distribution of the backscattered envelope. The variation of the Nakagami parameter from 0 to 1 corresponds to a change in the envelope statistics from pre-Rayleigh to Rayleigh distributions; a Nakagami parameter value higher than 1 indicates that the statistics of the backscattered signal conform to post-Rayleigh distributions.

The details for the initial model and hypothesis can be found in the previous study [26]. In brief, the Nakagami distribution model assumes that only one type of scatterer exists in the scattered media. However, the fat-infiltrated liver may be supposed as a mixture of hepatocytes, luminal structures, and lipid droplets. The percentage of hepatocytes is more than 80% of the entire volume of the liver, and the number of hepatocytes is much higher than those of the luminal structure. In this condition, the size and the number density of lipid droplets correlate with the grade of hepatic steatosis, and the increase in the lipid scatterers further results in the acoustic impedance mismatch to enhance the backscattering intensity and image brightness. This means that normal and fat-infiltrated hepatocytes (lipid droplets) are two primary scattering sources with different acoustic properties for fatty liver. The DND model combines two Nakagami distributions by using a weighted factor *α* to describe two scattering sources, as follows [26]:

(2) $$f_{mix}\left(x\right)=\left(1-\alpha \right)f_{L}(x|m_{L},\Omega_{L})+\alpha f_{F}(x|m_{F},\Omega_{F})$$

where *f_L_*(*m_L_*, Ω*_L_*) and *f_F_*(*m_F_*, Ω*_F_*) represent the PDFs obtained from normal liver tissue and lipid droplets, respectively. The parameter *m_L_* = 0.8 was used as a fixed priori according to the results obtained from rat experiments [26] and normal liver measurements [20]. Two methods are available to estimate the DND parameters (i.e., Ω*_L_*, *m_F_*, Ω*_F_*, and *α*): (i) optimization of Kullback–Leibler (KL) divergence and (ii) an expectation-maximization (EM) algorithm [26].

### 2.2. Clinical Subjects

This study was approved by the Institutional Review Board of the Chang Gung Memorial Hospital, Taiwan, from August 2017 to July 2020 (Approval No.: 201601928B0C501). All participants signed informed consent forms, and experimental methods were performed according to the approved guidelines. A total of 269 patients with confirmed chronic hepatitis B or C infection who were scheduled for a liver biopsy or partial liver resection were enrolled, and those with a history of liver resection, medications, abused alcohol, and focal hepatic steatosis were excluded from the data analysis. For the remaining subjects (*n* = 204), blood tests were conducted after 8 h of overnight fasting; thereafter, a clinical ultrasound imaging system (Model 3000, Terason, Burlington, MA, USA) equipped with a convex array transducer (Model 5C2A, Terason, Burlington, MA, USA) was used for ultrasound examination of the liver. The pulse length (PL) and the central frequency of the transducer were 2.3 mm and 3.5 MHz, respectively. The liver parenchyma was imaged using the intercostal scanning approach [27] (segment VIII; focus: 4 cm; depth: 8 cm) by the same experienced radiologist who was blinded to the medical histories of the study patients; five independent scans were performed to acquire image data consisting of 128 scan lines of backscattered RF signals at a sampling rate of 30 MHz. Following examination, liver resection or percutaneous liver biopsy of the same segment was performed within one week.

### 2.3. Ultrasound Data Processing

**Figure 1:**
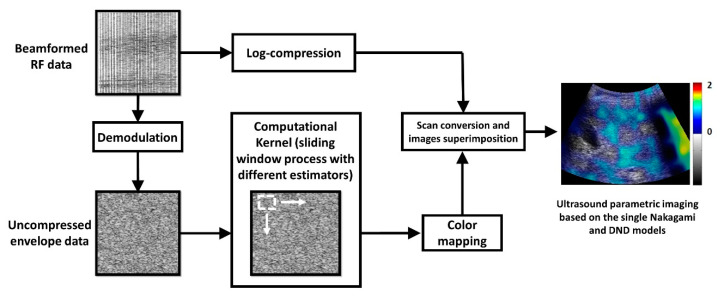
Illustration of the algorithm for ultrasound parametric imaging based on the single Nakagami and the double Nakagami distribution (DND) models. The sliding window was used to process the uncompressed envelope image, generating a parametric map to be superimposed on the B-mode image for the final display of the parametric image.

Figure 1 shows the algorithm for the parametric ultrasound imaging based on the single Nakagami and the DND models. For each set of raw image data, the absolute values of the Hilbert transform of each backscattered signal were calculated to obtain the envelope image, which was further compressed with the logarithmic transform of the B-mode imaging at the dynamic range of 40 dB. Concurrently, the uncompressed envelope image was processed using the sliding window technique [28,29,30] to construct ultrasound parametric images based on the conventional Nakagami (estimated using the statistical moments of backscattered envelopes [23,24,25], denoted as *m* image) and DND parameters estimated using the KL and EM methods (denoted as *m*_F(KL)_, and *m*_F(EM)_ images, respectively). The window overlap ratio was set at 50% to provide a tradeoff between the parametric image resolution and computational time [29]. The window side length (WSL) corresponding to three times the PL was used for *m* imaging [30]. The WSL was set at a varying range, from one to ten times the PL of the transducer, for clarifying an appropriate window size for DND imaging. A region of interest (ROI) was manually outlined on the B-mode image, and it was then applied to the corresponding *m*, *m*_F(KL)_, and *m*_F(EM)_ images to calculate the average of the pixel values (i.e., the *m*, *m*_F(KL)_, and *m*_F(EM)_ parameters) in the ROI.

The reason why the PL of the transducer was used as the unit to describe the WSL is explained below. Under using a convex array transducer, the sampling interval in the lateral axis for the image raw data obtained from the system is non-uniform. With increasing the distance from the transducer, the distance between each scan line increases. However, the axial sampling rate remains unchanged because it is determined by the analog-to-digital converter in the imaging system. For this reason, the axial length of the envelope signal is the primary factor to ensure descriptions of the signal waveform features and stable estimation of the statistical parameter [24]. Therefore, the PL of the transducer was recommended as the unit of the WSL [28,29].

### 2.4. Statistical Analysis

Hepatic steatosis grades identified according to histological findings were used for data grouping—normal (steatosis involving < 5% hepatocytes), mild (5–33%), moderate (33–66%), and severe (>66%) [31]. The liver fibrosis stage for each patient was also identified by the Metavir score; F0, no fibrosis; F1, portal fibrosis with no septa; F2, portal fibrosis with few septa; F3, bridging fibrosis with many septa; and F4, cirrhosis (nodular stage) [32]. The *m*, *m*_F(EM)_, and *m*_F(KL)_ parameters as a function of the grade are expressed as the median and interquartile range (IQR). The receiver operating characteristic (ROC) curve analysis with a 95% confidence interval (CI) was used to evaluate the diagnostic performance of the ultrasound Nakagami and DND imaging in the assessment of hepatic steatosis. The area under the ROC (AUROC) was used to determine the predictive value of the Nakagami and DND parameter for diagnosing each grade—normal versus ≥ mild, normal to mild versus ≥ moderate, and normal to moderate versus ≥ severe. The sensitivity, specificity, and accuracy were also determined. Furthermore, the *m*_F(EM)_ and *m*_F(KL)_ parameters were compared with the *m* parameter for performing curve fitting to confirm whether any differences existed between the conventional Nakagami and DND parameters. All statistical analyses were performed using SigmaPlot (version 12.0, Systat Software, Inc., San Jose, CA, USA).

## 3. Results

**Figure 2:**
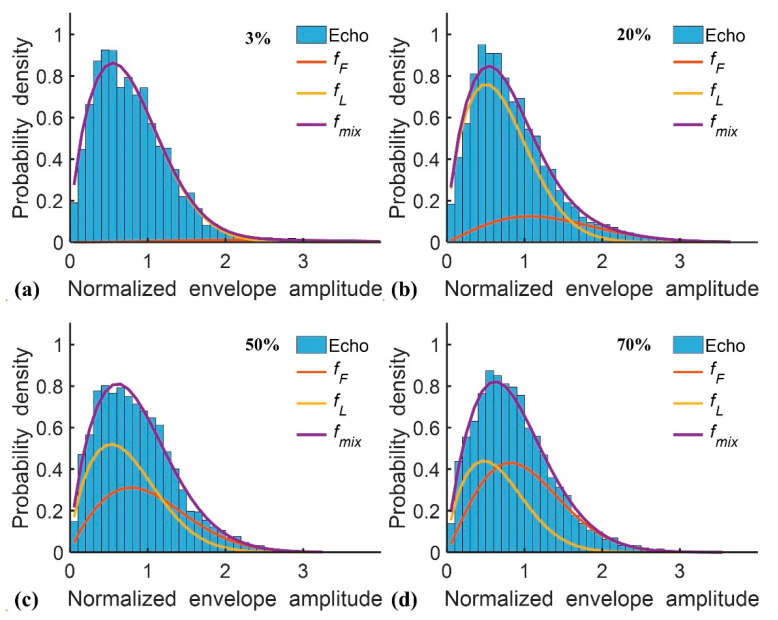
Probability density distributions of the DND mode constructed using the Kullback–Leibler (KL) method, representing that the backscattered signals contributed by lipid droplets (i.e., *f*_F_) gradually approach the Rayleigh distribution with an increasing grade of hepatic steatosis. (**a**) 3%; (**b**) 20%; (**c**) 50%; (**d**) 70%.

**Figure 3:**
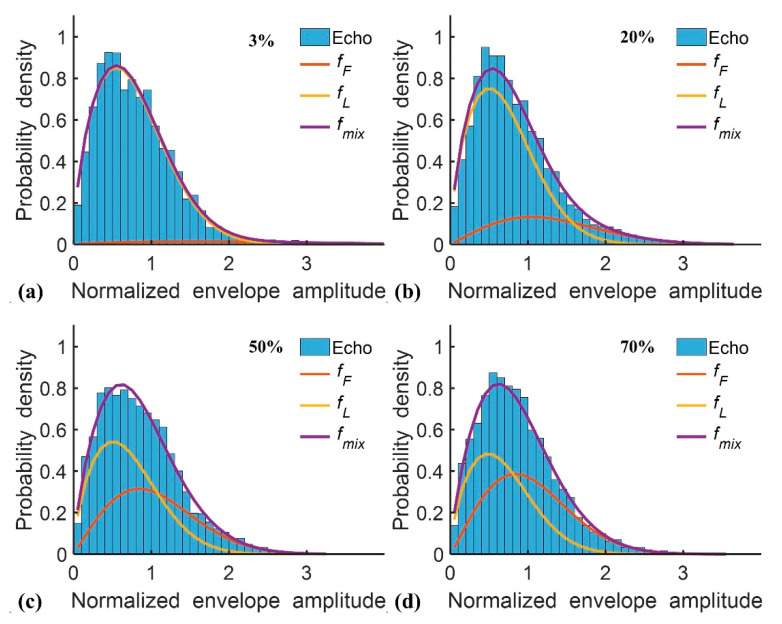
Probability density distributions of the DND mode constructed using the expectation-maximization (EM) method, representing that *f*_F_ varies with increasing grades of hepatic steatosis. (**a**) 3%; (**b**) 20%; (**c**) 50%; (**d**) 70%.

**Figure 4:**
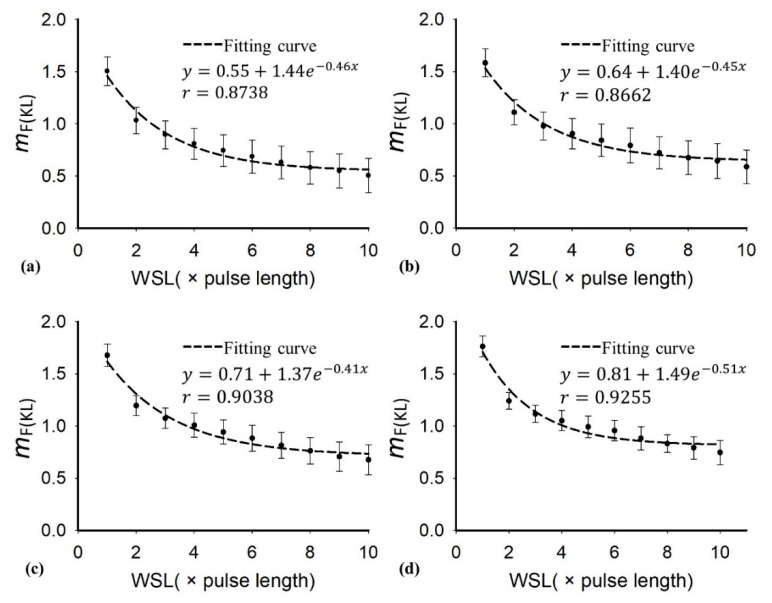
Average of the parameter *m*_F(KL)_ in the region of interest (ROI) as a function of the window side length (WSL) obtained with different grades of hepatic steatosis: (**a**) normal; (**b**) mild; (**c**) moderate; (**d**) severe. As the WSL increased, the parameter *m*_F(KL)_ gradually decreased to exhibit a relatively stable estimation.

**Figure 5:**
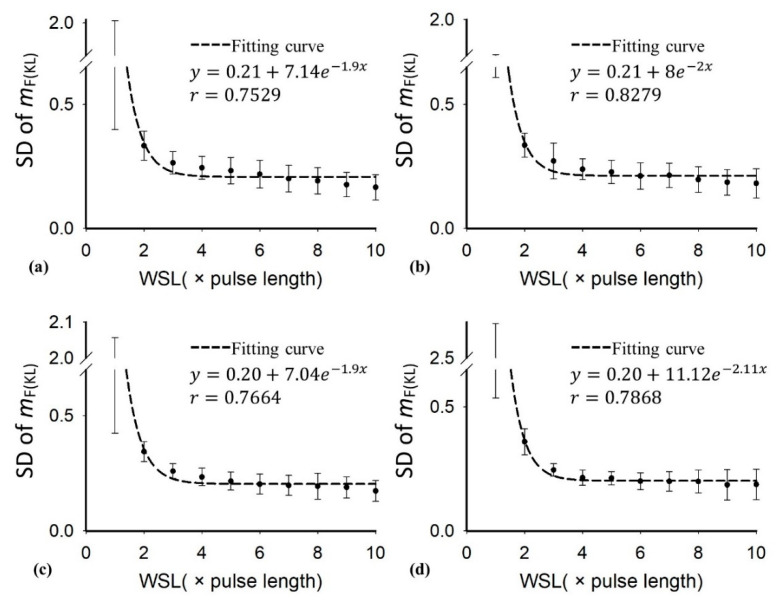
The average and standard deviation (SD) of the parameter *m*_F(KL)_ in the ROI as a function of the WSL obtained with different grades of hepatic steatosis: (**a**) normal; (**b**) mild; (**c**) moderate; (**d**) severe. By increasing the WSL, the SD of the parameter *m*_F(KL)_ gradually decreased, and the fitting curve plateaued when the WSL was larger than 5 PL.

**Figure 6:**
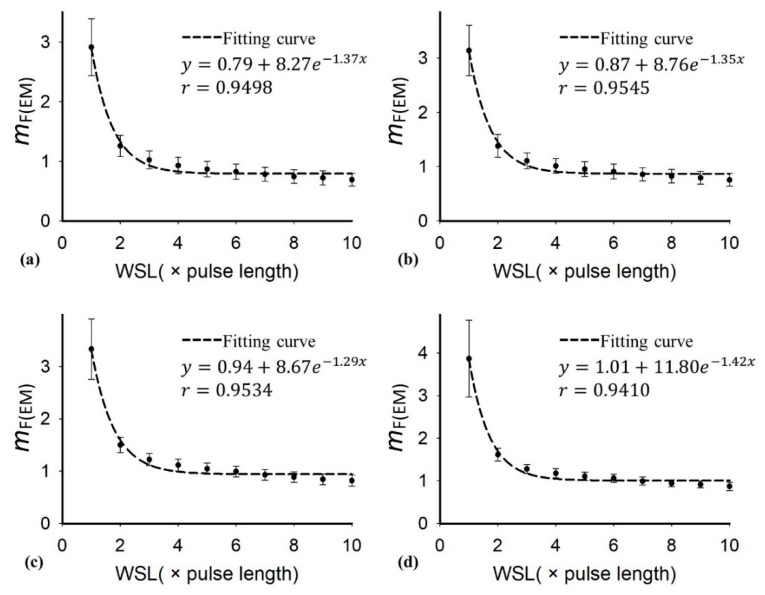
Average of the parameter *m*_F(EM)_ in the ROI as a function of the WSL obtained with different grades of hepatic steatosis: (**a**) normal; (**b**) mild; (**c**) moderate; (**d**) severe. As the WSL increased, the parameter *m*_F(EM)_ gradually decreased to exhibit a relatively stable estimation.

**Figure 7:**
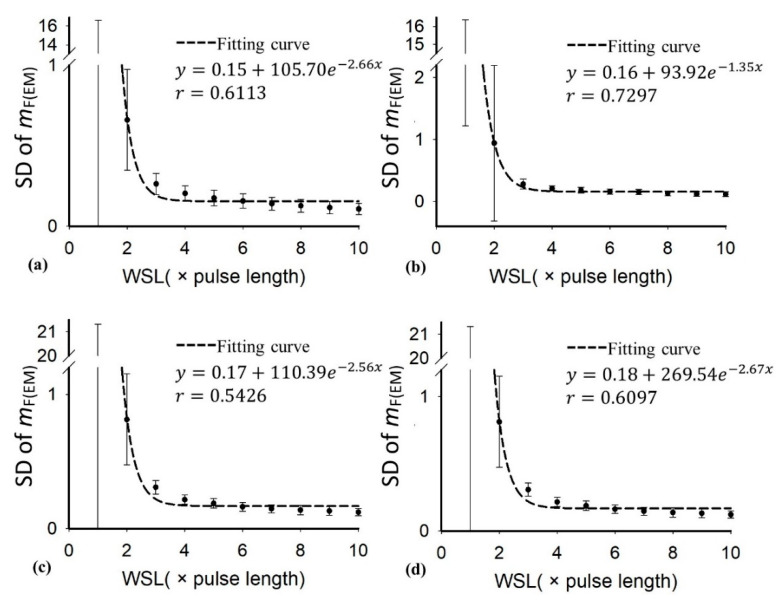
SD of the parameter *m*_F(EM)_ in the ROI as a function of the WSL obtained with different grades of hepatic steatosis: (**a**) normal; (**b**) mild; (**c**) moderate; (**d**) severe. By increasing the WSL, the SD of the parameter *m*_F(EM)_ gradually decreased, and the fitting curve plateaued when the WSL was larger than 5 PL.

**Figure 8:**
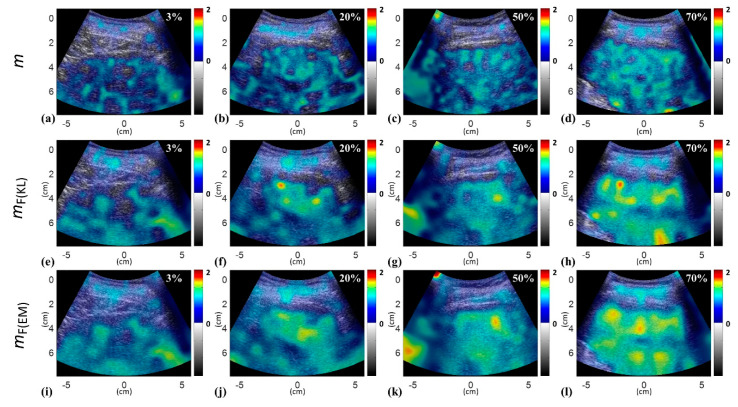
Ultrasound parametric imaging constructed using the *m* (**a**–**d**), *m*_F(KL)_ (**e**–**h**), and *m*_F(EM)_ (**i**–**l**) parameters for different grades of hepatic steatosis. The formation of hepatic steatosis strengthened the image brightness in the Nakagami and the proposed DND imaging, representing increases in the parameters *m*, *m*_F(KL)_, and *m*_F(EM)_.

Table 1 presents the demographic and blood test data of the recruited patients. Note that the subjects with hepatic steatosis also had different degrees of liver fibrosis; such a phenomenon is commonly seen in clinical cases. Figure 2 and Figure 3 applied to the KL and EM methods to reconstruct the PDFs of DND (i.e., *f*_mix_) and the components contributed by normal liver tissue (i.e., *f*_L_) and lipid droplets (i.e., *f*_F_) for the examples of different hepatic fat fractions (the backscattered envelope data were acquired from the ROIs corresponding to the liver parenchyma). The change in *f*_F_ from the pre-Rayleigh to Rayleigh distributions was observed when the degree of hepatic steatosis increased. To determine the appropriate WSL for constructing DND imaging using the KL and EM methods, the averages and standard deviation (SD) of *m*_F(KL)_ and *m*_F(EM)_ with different WSLs for each hepatic steatosis grade were explored (Figure 4, Figure 5, Figure 6 and Figure 7). As the WSL was increased from one to ten times the PL, the average *m*_F(KL)_ gradually decreased and became relatively stable. Concurrently, the SD of *m*_F(KL)_ also decreased, and the fitting curve plateaued when the WSL was more than five times the PL. These observations were also found for *m*_F(EM)_, implying that WSL that is five times the PL is appropriate for ultrasound DND imaging. Figure 8 depicts typical parametric images of *m*, *m*_F(KL)_, and *m*_F(EM)_ for different hepatic fat fractions. Hepatic steatosis strengthened the brightness in the Nakagami and the proposed DND images, representing increases in the parameters of *m*, *m*_F(KL)_, and *m*_F(EM)_. The *m*_F(KL)_ and *m*_F(EM)_ images appeared to be brighter than the *m* image, indicating that the *m*_F(KL)_ and *m*_F(EM)_ parameters obtained from the DND model are higher than those of the single Nakagami distribution.

**Figure 9:**
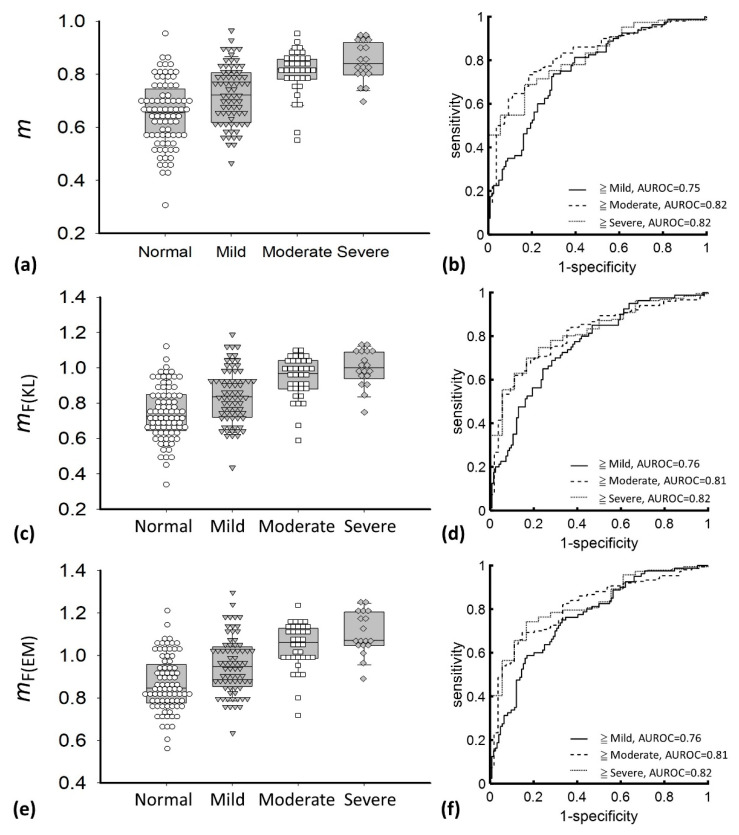
Parameters *m*, *m*_F(KL)_, and *m*_F(EM)_ as a function of hepatic steatosis grade (**a**–**c**) and the ROC curves for diagnosing hepatic steatosis (**d**–**f**). Ultrasound DND imaging was able to detect hepatic steatosis with the same performance as the conventional Nakagami imaging.

**Figure 10:**
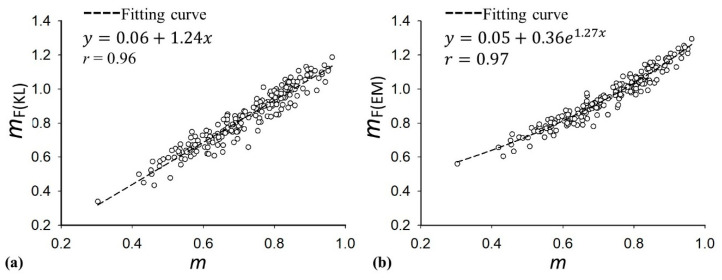
Comparisons of the *m*_F(KL)_ and *m*_F(EM)_ parameters with the *m* parameter. (**a**) *m*_F(KL)_; (**b**) *m*_F(EM)_. The linear and exponential increasing functions were used for performing curve fitting. A nonlinear relationship between the *m*_F(EM)_ and *m* parameters was found.

Figure 9 displays the parameters of *m*, *m*_F(KL)_, and *m*_F(EM)_ as a function of the hepatic steatosis grade and the ROC curves for diagnosing different grades of hepatic steatosis. The median values of *m* were 0.65 (IQR: 0.56–0.73) for normal, 0.72 (IQR: 0.61–0.80) for mild, 0.82 (IQR: 0.78–0.85) for moderate, and 0.83 (IQR: 0.80–0.91) for severe hepatic steatosis. The median values of *m*_F(KL)_ for normal, mild, moderate, and severe hepatic steatosis were 0.72 (IQR: 0.64–0.84), 0.83 (IQR: 0.72-0.93), 0.96 (IQR: 0.88–1.04), and 1.00 (IQR: 0.94–1.08), respectively, and those of *m*_F(EM)_ were 0.84 (IQR: 0.77–0.95), 0.94 (IQR: 0.85–1.03), 1.06 (IQR: 0.98–1.12), and 1.07 (IQR: 1.04–1.20), respectively. The AUROCs of *m*, *m*_F(KL)_, and *m*_F(EM)_ in diagnosing steatosis grades of ≥mild were 0.75 (95% CI: 0.69–0.83), 0.76 (95% CI: 0.69–0.83), and 0.76 (95% CI: 0.69–0.83), respectively; those of ≥moderate were 0.82 (95% CI: 0.77–0.88), 0.81 (95% CI: 0.75–0.87), and 0.81 (95% CI: 0.75–0.87), respectively, and those of ≥severe were 0.82 (95% CI: 0.74–0.90), 0.82 (95% CI: 0.74–0.90), and 0.82 (95% CI: 0.74–0.90), respectively. Table 2 summarizes the diagnostic performance of *m*, *m*_F(EM)_, and *m*_F(KL)_ parametric imaging in the grading of hepatic steatosis. Figure 10 shows comparisons of the *m*_F(KL)_ and *m*_F(EM)_ parameters with the *m* parameter, indicating that the DND parameter is proportional to the conventional Nakagami parameter. To more precisely describe the relationships between the single Nakagami and the DND parameters, the equations that are available in SigmaPlot were used to fit the data, and the linear and exponential increasing functions were found to maximize the correlation coefficients *r* of *m* with *m*_F(KL)_ (*r* = 0.96) and *m*_F(EM)_ (*r* = 0.97), respectively. Interestingly, a nonlinear relationship existed between the *m*_F(EM)_ and *m* parameters.

## 4. Discussion

### 4.1. Significance of this Study

The DND model is a novel Nakagami statistics-based approach that was initially developed for using high-frequency ultrasound to describe the backscattered signals measured from fat-infiltrated liver tissues. Prior to this study, no reports and literature explored the optimal window size, estimation methods, and computational procedures for clinical-oriented DND imaging. This study standardized the algorithmic scheme and confirmed the feasibility of the DND model in the clinical assessment of hepatic steatosis using low-frequency ultrasound dedicated to abdominal examinations. To provide stable and reliable DND parameter estimations, an appropriate WSL for using the sliding window technique to construct ultrasound DND imaging was suggested as five times the PL. Using both the KL and EM methods, the DND parameters could be estimated to allow imaging and grading of hepatic steatosis with a promising diagnostic performance. This is the first to clinically reveal the usefulness of ultrasound DND imaging in diagnosing hepatic steatosis.

### 4.2. The Dependency of the DND Parameter on Hepatic Steatosis

Compared with the conventional Nakagami parameter, the DND parameter specifically reflects the statistical distribution of ultrasound signals backscattered from fat-infiltrated hepatocytes in the liver parenchyma [26]. The effects of hepatic steatosis on the Nakagami parameter have been discussed by modeling the liver tissue as a scattering medium [23,24,25]; the same concept could be used to explain how the DND parameter varies with the degree of hepatic steatosis. In brief, with increasing grades of hepatic steatosis, the number of lipid droplets increases; thus, this condition could be identified as a process of increase in the number densities of the scatterers, which alters the backscattered statistics to develop toward the Rayleigh distribution [25]. This may explain why the DND parameter increases with the severity of hepatic steatosis. Note that liver fibrosis tends to result in a relatively high degree of variance in the scattering cross-sections of the scatterers, making the backscattered statistics change toward the pre-Rayleigh distribution (decreasing the Nakagami parameter) [30]. Consequently, the confounding effects (due to liver fibrosis and hepatic steatosis) on the backscattered statistics existing in the current dataset may influence the dependency of the Nakagami and the DND parameter on hepatic steatosis.

### 4.3. Comparisons between the Conventional Nakagami and DND Parameters

It has been shown that the DND model can fit the envelope statistics relatively accurately in cases of small and large numbers of lipid droplets; this advantage over the single Nakagami distribution is attributed to the increase in the number of degrees of freedom in the data analysis [26]. However, the estimated parameter of the DND model is typically higher than that obtained using the conventional Nakagami distribution [26]. This phenomenon was also found in the current clinical results. This may be explained on the basis of the difference between the single Nakagami and the DND model. The backscattering information contributed by the liver parenchyma (including hepatocytes and luminal structures) and lipid droplets are considered independently in the DND model. Some information contributed from the structural components that tend to result in the backscattered statistics of the pre-Rayleigh distribution could be eliminated when estimating the DND parameter. In other words, using the conventional Nakagami parameter of the Nakagami distribution may underestimate the equivalent scatterer concentration corresponding to hepatic steatosis; in comparison, the curve fitting analysis revealed the nonlinear relationship between the *m*_F(EM)_ and *m* parameters, demonstrating that the DND parameter essentially differs from the single Nakagami parameter. Although the diagnostic performance of the DND imaging was the same as that of the conventional Nakagami imaging, the physical interpretations given by the DND model is more specific to steatosis information, and the EM method was recommended to compensate the effect of the parameter underestimation in conventional Nakagami imaging by satisfying the nonlinear dependency of the DND parameter on the *m* parameter.

### 4.4. Limitations and Future Work

This study has some limitations. The DND parameter measured using a high-frequency (14.4 MHz) ultrasound provided sensitive detection of hepatic steatosis in rats [26]. Comparatively, the clinical performance of DND imaging constructed using low-frequency ultrasound was not significantly different from that of conventional Nakagami imaging. Probably, high-frequency ultrasound is a key factor to endow DND imaging with improved sensitivity to variations in the scatterer properties, implying that the DND model used clinically needs optimization or modification. The other challenge would be how we determine the parameter *m_L_* more accurately for estimating the DND parameters. Different subjects may have different *m_L_* values because of the differences in tissue microstructures between individuals. During ultrasound DND parametric imaging, each window location may also correspond to different *m_L_* values. This problem is indeed hard to conclude at the current stage, and using a constant *m_L_* as the prior condition may be temporally a solution. Moreover, the DND model requires a larger window size (WSL = 5 PL) for acquiring more envelope data to satisfy the requirements of the estimation methods. However, the conventional Nakagami parameters based on moment estimation require only WSL = 3 PL to support parametric imaging [30]. Prior to translating the DND model from animals to clinical applications, the resolution enhancement may be the priority for future work, and intra- and interoperator reliabilities also require further investigation.

## 5. Conclusions

This study demonstrated the clinical feasibility of parametric imaging based on the DND model in the assessment of hepatic steatosis using low-frequency ultrasound. DND imaging constructed using the sliding window with the WSL = 5 PL provided stable and reliable parameter estimations and image visualization, endowing the DND parameter with the ability to grade hepatic steatosis and reflect backscattering information associated with lipid droplets, which benefit interpretations of the nature of the fat-infiltrated liver. Sensitivity improvement, resolution enhancement, and intra- and interoperator reliabilities for ultrasound DND imaging require further investigations.

## Figures and Tables

**Table 1:** Demographic data of patients enrolled in the study.

Characteristics	Value
**Male/Female**	129/75
**Age, years**	
Mean ± standard deviation (range)	57.75 ± 11.08 (31–81)
Median	58
**BMI, kg/m^2^**	
Mean ± standard deviation (range)	25.38± 3.91 (16.82–37.83)
Median	24.91
**AST, U/L**	
Mean ± standard deviation (range)	67.39 ± 68.04 (15–507)
Median	46
**ALT, U/L**	
Mean ± standard deviation (range)	87.64 ± 99.33 (8–595)
Median	53
**Histological grade, no. of patients**	
Normal	80
Mild	70
Moderate	36
Severe	18
**Metavir score, no. of patients**	
F0	16
F1	40
F2	46
F3	61
F4	41

Note—Unless otherwise noted, data are numbers of patients. BMI: body mass index, AST: aspartate aminotransferase, ALT: alanine aminotransferase, Normal AST levels for female and male subjects are less than 35 U/L and 50 U/L, respectively. Normal ALT levels for female and male subjects are less than 19 U/L and 30 U/L, respectively.

**Table 2:** Comparisons of ROC analysis for using DND imaging to diagnose different grades of hepatic steatosis.

Parameter	*m*			*m*_F(KL)_			*m*_F(EM)_		
	≥ Mild	≥ Moderate	≥ Severe	≥ Mild	≥ Moderate	≥ Severe	≥ Mild	≥ Moderate	≥ Severe
Cutoff value	0.71	0.77	0.79	0.80	0.88	0.94	0.96	1.03	1.04
Sensitivity, %	73.75	73.33	68.82	68.75	69.33	74.73	68.75	71.33	68.28
Specificity, %	70.16	81.18	83.33	71.77	81.48	77.78	75	77.78	83.33
LR+	2.47	3.96	4.13	2.44	3.74	3.36	2.75	3.21	4.10
LR−	0.37	0.33	0.37	0.44	0.38	0.32	0.42	0.37	0.38
PPV, %	61.46	91.67	97.71	61.11	91.23	97.20	63.95	89.92	97.69
NPV, %	80.56	52.38	20.55	78.07	48.89	22.95	78.81	49.41	20.27
AUROC (95% CI)	0.75 (0.69–0.83)	0.82 (0.77–0.88)	0.82 (0.74–0.90)	0.76 (0.69–0.83)	0.81 (0.75–0.87)	0.82 (0.74–0.90)	0.76 (0.69–0.83)	0.81 (0.75–0.87)	0.82 (0.74–0.90)
